# Marine predators algorithm for solving single-objective optimal power flow

**DOI:** 10.1371/journal.pone.0256050

**Published:** 2021-08-12

**Authors:** Mohammad Zohrul Islam, Mohammad Lutfi Othman, Noor Izzri Abdul Wahab, Veerapandiyan Veerasamy, Saifur Rahman Opu, Abinaya Inbamani, Vishalakshi Annamalai

**Affiliations:** 1 Advanced Lightning, Power and Energy Research (ALPER), Department of Electrical and Electronic Engineering, Faculty of Engineering, Universiti Putra Malaysia (UPM), UPM Serdang, Seri Kembangan, Selangor, Malaysia; 2 Department of Electrical and Electronic Engineering, Leading University, Sylhet, Bangladesh; 3 Department of Electrical and Electronic Engineering, Sri Ramakrishna Engineering College, Coimbatore, India; J.C. Bose University of Science and Technology, YMCA, INDIA

## Abstract

This study presents a nature-inspired, and metaheuristic-based Marine predator algorithm (MPA) for solving the optimal power flow (OPF) problem. The significant insight of MPA is the widespread foraging strategy called the Levy walk and Brownian movements in ocean predators, including the optimal encounter rate policy in biological interaction among predators and prey which make the method to solve the real-world engineering problems of OPF. The OPF problem has been extensively used in power system operation, planning, and management over a long time. In this work, the MPA is analyzed to solve the single-objective OPF problem considering the fuel cost, real and reactive power loss, voltage deviation, and voltage stability enhancement index as objective functions. The proposed method is tested on IEEE 30-bus test system and the obtained results by the proposed method are compared with recent literature studies. The acquired results demonstrate that the proposed method is quite competitive among the nature-inspired optimization techniques reported in the literature.

## Introduction

The optimal power flow (OPF) is an inevitable part of the energy management system for power system planning and operation over a couple of decades. The main objective of the OPF is to determine the most favourable operating conditions to meet the required demand by satisfying all the power system operational and security constraints [1]. In 1960, French scholar Carpentier proposed the concept of OPF to ensure reliable and economic power generation based on precise mathematics [2]. In this context, several selective objective functions, for instance, total generation cost, real/reactive power loss, and voltage deviation have been considered to obtain the optimal dispatch of generation by different numerical and artificial intelligence (AI) techniques [3]. Generally, the OPF problem is a static non-linear, non-convex, large scale, and highly constrained optimization problem in power system networks that deal with a set of independent and state variables. The control variables are the generator real power, generator bus voltages, reactive power injections of VAR compensators, and transformer tap settings while the state variables including the generator reactive power, load bus voltages, and the transmission lines limit [4]. Recently, the ever-increasing energy demand introduces a massive challenge to the prevailing networks to deliver quality power to the consumer end efficiently and economically [5]. Therefore, power utilities were repeatedly exploring several economic operational strategies in the power generation of power by enforcing equality and inequality constraints to deliver uninterrupted power supply [6]. Moreover, due to the ever-increasing power demand, the modern power system has been operating close to its power transfer capability limit that leads to stressed conditions of the system. Occasionally, a small change in the operating conditions results in system instability due to a dip in the voltage level that may cause blackouts or brownouts of the system as similar events have been witnessed in North America, Canada, India, Pakistan, and so on over the last few decades [7, 8] Therefore, solving the OPF problem is most important to assess the voltage stability of the system.

Numerous optimization techniques have been employed to solve the OPF problems with different selective objective functions of generation cost, power loss, environmental emission, voltage deviation, and voltage stability assessment index. However, most of the work in the literature attempted to solve the OPF problem to minimize the power loss for the given operating loads. In general, the techniques to solve the OPF problem can be categorized into classical and heuristic-based techniques. The classical method includes the Newton method, gradient method, interior point method, linear programming, and non-linear programming [9]. These techniques were introduced with different theoretical assumptions of convexity, differentiability, and continuity which are not relevant to solve the OPF problems. Further, the convergence of all the classical methods is immensely gambled on the initial guess [10] and these also endure acute limitations in dealing with non-linear, discrete-continuous functions and control variables [11]. Moreover, the solution quality deteriorates when the number of the controlling parameters increases [12].

To overcome the aforementioned drawbacks of classical methods, researchers have proposed nature-inspired heuristic-based optimization techniques for solving the OPF problem due to the tremendous development of computer technology [13]. These techniques can be broadly categorized into evolutionary-based, swarm-based, physics-based, and human‐based algorithms [14]. Due to the easy implementation and effectiveness in securing the global optimality, many heuristic-based techniques have been employed to solve OPF problems considering various objective functions in the power system [15]. Kwang Y. Lee Xiaomin Bai [16], presented a modified version of the conventional genetic algorithm (GA) to deal with OPF problems in the power system. The main goal of this study was to reduce the reactive power loss of the system and the obtained results were compared with successive linear programming. In [17], the load flow and the economic dispatch problem were considered to verify the viability of using GA to solve the OPF problems. Xiaohui Yuan et al., have proposed an improved Pareto evolutionary algorithm to solve OPF problems considering fuel cost and emission as objective functions [9]. A Biogeography- based Optimization (BBO) technique has been used to solve several objective functions as a single-objective OPF problem by A. Bhattacharya et al. [18]. Similarly, physics-based optimization techniques namely Big-Bang Big Crunch Algorithm (BBBC) [19], Gravitational Search Algorithm (GSA) [20] were applied to solve the OPF problem. Moreover, many researchers have also employed several human-based techniques in solving OPF problems. Based on the influence of a teacher on learners, Teaching-Learning-Based Optimization (TLBO) [21], Harmony Search Algorithm (HS) [22], Tabu Search Algorithm (TS) [23] were used to deal with the constrained OPF problems to get a better optimal solution. In some cases, these techniques demonstrate promising results but stuck in local optima. Hence, several swarming behaviour-based techniques got attention for solving OPF problems in the literature. A Particle Swarm Optimization (PSO) [24] was proposed to solve OPF problems including fuel cost minimization, voltage profile improvement, and voltage stability enhancement. Further, some meta-heuristic based techniques, for example, Whale Optimization Algorithm (WOA) [25], Moth-Flame Optimization Algorithm (MFO) [26], Glowworm Swarm Optimization Algorithm (GSO) [27], Jaya Algorithm (JA) [28], Artificial Bee Colony Algorithm (ABC) [29] were employed to solve OPF problem effectively and accurately. Lately, a hybrid self-adaptive heuristic algorithm was used to solve OPF problems considering the total fuel cost, active power losses, and the emission in [30]. Based on the trophy-winning behaviour of players, the most valuable player algorithm (MVPA) belonging to the family of swarm intelligence was proposed by Koganti Srilakshmi et al., for solving OPF problems on several bus test systems [31]. On the other hand, the authors in [32] proposes a Turbulent flow of water-based optimization using the concept of nature search phenomenon to solve the economic load dispatch problem of fuel cost minimization considering the effects of valve points and ramp rate limits. A multi-objective backtracking search algorithm has been proposed to solve the disparate combinations of multi-objective (fuel cost, power loss, voltage deviations) OPF for IEEE 57-bus and 118-bus system [33]. Several other optimization approaches of phasor based PSO, improved wind driven algorithm and adaptive quasi-oppositional differential evaluation algorithm with migration operator of BBO were proposed to enhance the exploration and exploitation search ability of agents to reach the global minima in order to solve the different combinations of OPF problems [34–36]. However, As the rule of thumb states that all the optimization techniques proposed in literature do not provide optimal solutions for all kind of engineering optimization problems. Because, each technique has certain limitations to solve the particular type of problems like their own merits and demerits to solve OPF problems. Therefore, researchers continuously were looking for powerful nature-inspired optimization techniques to solve the OPF problems. In view of this, a recently developed optimization technique has been used to solve the OPF problem because of its distinct foraging strategy and Brownian movements as well as the biological interaction between predators and prey to get the optimal solution. The prime contributions of this paper are as follows:

- Solving single-objective OPF problem using MPA technique to minimize the fuel cost, real power loss, reactive power losses, voltage deviation and voltage stability index of the power system.
- The effectiveness of the method is tested on the IEEE 30-bus test system for different selective single objectives by satisfying the equality and inequality constraints of the network.
- The result obtained is compared with other well-known optimization techniques presented in recent literary works.
- The robustness of the proposed MPA based OPF method is validated for large-scale power system of IEEE 118-bus system.

The remainder of the paper is organized as follows: Section 2 deals with the OPF problem formulation which describes the various single-objective problem formation mathematically including equality and inequality constraints. While section 3 presents the proposed intelligence-based MPA technique with a dynamic levy flight strategy. The results and discussion of the proposed method technique with other well-known nature-inspired methods of optimization are is presented in section 4. Finally, section 5 portrays the conclusion and future scope of the work.

## 2. OPF problem formulation

This section presents the mathematical formulation of OPF and different selective objectives for the smooth and reliable operation of power networks. The OPF is a highly non-linear, non-convex and constrained optimization problem. The optimal power flow problem can be solved as a single or multi- objective function while satisfying equality and inequality constraints. In many research works, several objectives, for instance, fuel cost, real power loss, environment emission, voltage stability improvement have been considered individually or collectively that will be either maximized or minimized. In terms of optimization of real power generation, the generator bus voltage, reactive power compensator and transformer tap settings are the principles controlling parameters.

### 2.1 Single objective function

The objective function to be minimized is defined as,

Optimize, f_i_ (x, u) i = 1, 2, 3,…,N

Subject to equality and inequality constraints represented as,

g_j_ (x, u) = 0 j = 1, 2, 3,…,N

h_k_ (x, u)≤0 k = 1, 2, 3,…,N where, f is the i^th^ objective function, N denotes the total number of objective functions, u and x are the control and dependent variable, respectively, g_j_ and h_k_ are the equality and inequality constraints in j^th^ and k^th^ limits. The control variable u can be stated as,

u = [P_G2,…,GN_, V_G1,…,GN_, Q_cap1,…,capN_, T_1,…,N_] where, P_G2,…GN_ denotes the real power generation of N generators except the slack bus, V_G1,…GN_ represents voltage magnitude of generator bus, Q_cap1,…capN_ depicts the shunt VAR compensator and T_1,…,N_ is the tap settings of transformers.

On the other hand, the vector of dependent variable x can be represented as,

x = [P_Gslack_, V_L1…LN_, Q_G1…GN_, S_L1…LN_] where,

P_Gslack_, denotes the real power generation of slack bus,

V_L1…LN_ is the voltage magnitude of all load buses,

Q_G1…GN_, is the reactive power generation, and

S_L1,…LN_ is the transmission line capacity limit.

The various selective objective functions considered in this work are as follows:

### 2.2 Fuel cost minimization

In general, most of the literature work is based on fuel cost minimization as utility requires to generate electricity with the least cost by considering the deregulation and open market policy. The fuel cost function can be represented as a quadratic function of real power generations of generators which can be mathematically defined as,

$$f_{1}=\sum_{i=1}^{NG}a_{i}+b_{i}P_{Gi}+c_{i}\;P_{Gi}{}^{2}\;\$/h\;i=1,2,\ldots ,N_{G}$$ (1)

where, P_Gi_ is the total power generation in MW, a_i_, b_i_, and c_i_ denote the cost co-efficient of the specific generator, and N_G_ is the total number of generators in the system.

### 2.3 Active power loss minimization (APL)

To enhance the power quality to the consumer end, the APL is considered as an objective function which can be optimized by tuning the controlling parameters of the system by satisfying the power flow constraints. Mathematically, APL can be described as,

$$f_{2}=\sum_{i=1}^{NL}g_{i}[V_{k}{}^{2}+V_{m}{}^{2}-2V_{k}V_{m}\cos(\delta_{k}-\delta_{m})]\;\mathrm{MW}\;i=1,\ldots ,N_{L}$$ (2)

where, g_i_ is the transfer conductance, V_k_ and V_m_ represent the voltage magnitude of from and to buses, respectively, δ_k_ and δ_m_ depicts the phase angle, and N_L_ is the total number of transmission lines of the system.

### 2.4 Reactive power loss minimization (RPL)

To ensure a reliable power supply with balanced voltage, another significant factor of reactive power loss need to consider as an objective function. The RPL can be optimized by tuning the controlling parameters of the system by satisfying power flow constraints. The mathematical formulation of RPL is as follows,

$$f_{3}=\sum_{i=1}^{NL}g_{i}[{V_{k}}^{2}+{V_{m}}^{2}-2V_{k}V_{m}cos(\delta_{k}-\delta_{m})]\;MVari=1,\ldots ,N_{L}$$ (3)

### 2.5 Voltage deviation (VD)

Generally, the voltage deviation range lies between ±5% of nominal values to ensure the stable operation of the system. Mostly in the power network, the voltage magnitude at the bus should be maintained at 1 p. u. However, the deviation in bus voltage occurs due to a sudden increase in load demand, insufficient reactive power support, fault or any interruption may happen. Therefore, voltage deviation is considered to minimize and can be expressed as,

$$f_{4}=\sum_{i=1}^{NG}|V_{i}-1|\;i=1,2,3,4,\ldots ,NG$$ (4)

### 2.6 Voltage stability enhancement index

In addition to the fuel cost and loss function, this paper also considers the voltage stability index to assess the system stability. The voltage stability enhancement index (VSEI) is formulated as the sum of squared L-index for a given system operating condition, and is formulated as,

$${Minimize,\;f}_{5}=f_{VSI}=max\;(L_{f})$$ (5)

where, the L-index gives the proximity of the system to voltage collapse and can be defined as,

$$L_{f}=\left[1-\sum_{i=1}^{N_{G}}F_{ji}\frac{V_{i}}{V_{j}}\right]$$ (6)

where, Fij is a matrix generated from Y-bus while Vi and Vj are the voltage magnitude at i and j bus, respectively.

### 2.7 Equality constraints

The active and reactive power flow balance equation between the generated and absorbed power are generally referred to the equality constraints. These restrictions are one of the most important controlling parameters in the power system, while the load demands need to be satisfied by the generation. The equality constraints are defined as follows,

$$P_{i}(V,\delta )‐P_{\mathrm{Gi}}+P_{\mathrm{Di}}=0\;(i=1,2,3,\ldots ,N)$$ (7)

$$Q_{i}(V,\delta )‐Q_{Gi}+Q_{Di}=0\;(i=1,2,3,\ldots ,N)$$ (8)

where, P_i_ (V, δ) and Q_i_ (V, δ) are the real and reactive power flow equations and can be defined as,

$$P_{i}(V,\delta )=V_{i}\sum_{j=1}^{n}V_{j}(H_{ij}cos(\delta_{i}‐\delta_{j})+M_{ij}sin(\delta_{i}‐\delta_{j})$$ (9)

$$Q_{i}(V,\delta )=V_{i}\sum_{j=1}^{n}V_{j}(H_{ij}sin(\delta_{i}‐\delta_{j})‐M_{ij}cos(\delta_{i}‐\delta_{j})$$ (10)

$$\sum_{i=1}^{NG}P_{Gi}=P_{Di}+P_{loss}$$ (11)

where, N_G_ is the number of generator buses, N represents the number of bus, P_i_ depicts the active power injection, Q_i_ denotes the reactive power injection, P_Di_ represents the active power load demand, Q_Di_ is the reactive power load demand, P_Gi_ is the active power generation, Q_Gi_ is the reactive power generation, V is the voltage magnitude in p.u, δ is the phase angle in rad, the admittance matrix can be defined as Y_ij_ = H_ij_+jM_ij_, i and j are the from and to buses, and P_loss_ is the active power loss.

### 2.8 Inequality constraints

The inequality constraints are also called power system operating and security constraints which include the power generation limit of generating units, voltage magnitude of generator bus, transformer tap settings, and so on. These constraints are discussed as follows,

### 2.9 Generator constraints

The power generation and voltage limit can be expressed as follows for economic and reliable operation of the power system:

$$P_{Gimin}\leq P_{Gi}\leq P_{Gimax}\;(i=1,2,3,\ldots ,N_{G})$$ (12)

$$Q_{Gimin}\leq Q_{Gi}\leq Q_{Gimax}\;(i=1,2,3,\ldots ,N_{G})$$ (13)

$$V_{imin}\leq V_{i}\leq V_{imax}\;(i=1,2,3,\ldots ,N_{G})$$ (14)

### 2.10 Transformer constraints

The tap changing transformers in the power system is used to control the voltage magnitudes at a given bus to maintain the operational limits. The tap of transformers can be modelled in terms of a reactive power source which can be represented by,

$$T_{imin}\leq T_{i}\leq T_{imax}\;(i=1,2,3,\ldots ,N_{T})$$ (15)

### 2.11 Shunt compensator VAR constraints

Shunt compensator is used to maintain the voltage at the prescribed limit in order to improve the power factor. The system voltage can be maintained at the specified range by adding shunt or series reactors. The switchable shunt compensation can be designated to operate within the limit as follows,

$$Q_{imin}\leq Q_{i}\leq Q_{imax}\;(i=1,2,3,\ldots ,N_{G})$$ (16)

### 2.12 Security constraints

Overhead lines absorb reactive power when it is fully loaded. The long transmission lines with light load act as reactive power generators due to the predominance of the line capacitance. In addition, the voltage magnitude of the healthy power system should be within the range of V_Limin_ to V_Limax_ as follows,

$$V_{Limin}\leq V_{Li}\leq V_{Limax}\;(i=1,2,3,\ldots ,N)$$ (17)

$$S_{Limin}\leq S_{Li}\leq S_{Limax}\;(i=1,2,3,\ldots ,N)$$ (18)

## 3. Application of MPA to OPF problem

MPA is a population-based meta-heuristic optimization technique proposed by Afshin Faramarzi [37]. The detailed steps for MPA based optimization are presented as follows:

### 3.1 MPA formulation

Like other population-based methods, the initial solution in MPA is uniformly distributed over the search region in the first iteration as follows:

$$X_{0}=X_{min}+rand(X_{max}-X_{min})$$ (19)

where, X_min_ and X_max_ denote the lower and upper limit of control variables, respectively, and the rand is a random value in the range of (0, 1). According to the survival of the fittest theory, the top predators in nature are more talented in foraging. Therefore, the fittest solution is considered as a top predator to develop a matrix called Elite. The elements of this matrix can be used to find the prey based on the information of prey’s positions and which can be defined as:

$$\mathrm{Elite}={\left[\begin{array}{cccc} X_{1.1}^{1} & X_{1.2}^{1} & \ldots & X_{1.d}^{1}\\ X_{2.1}^{1} & X_{2.2}^{1} & \ldots & X_{2.d}^{1}\\ \vdots & \vdots & \vdots & \vdots \\ X_{n.1}^{1} & X_{n.2}^{1} & \ldots & X_{n.d}^{1} \end{array}\right]}_{n\times d}$$ (20)

where $\vec{X^{1}}$ represents the top predator vector, n is the number of search agents, and d is the number of dimensions. Both predator and prey are looking for their own food and are considered as the search agents. At the end of every iteration, the Elite matrix is updated by the better predator compared to the top predator in its previous iteration.

Another matrix is called prey which is framed with the same dimension as that of the Elite matrix. Generally, during the initialization process, the prey is constructed in which the predators update their position. Among the initial prey, the fittest one is used to construct the Elite matrix. The Prey matrix is presented as:

$$\mathrm{Prey}={\left[\begin{array}{cccc} X_{1.1} & X_{1.2} & \ldots & X_{1.d}\\ X_{2.1} & X_{2.2} & \ldots & X_{2.d}\\ \vdots & \vdots & \vdots & \vdots \\ X_{n.1} & X_{n.2} & \ldots & X_{n.d} \end{array}\right]}_{n\times d}$$ (21)

where, X_i,j_ represents the j^th^ dimension of i^th^ prey. The entire optimization process is mainly depending on the above specified two matrices.

### 3.2 MPA optimization scenarios

On considering the velocity ratio and mimicking pattern of predator and prey, the whole MPA optimization process can be categorized into three main phases. The various phases that occur based on the velocity of movement of prey to escape from predators are: high-velocity ratio, unit velocity ratio, and low-velocity ratio phases. In MPA, each phase is specified and assigned with a particular period of iteration. These phases are defined based on the rules overseen on the nature of predator and prey movement while mimicking it. The following phases are described in detail as follows:

Phase 1: During this phase, the prey is moving faster than the predator with a high-velocity ratio. This phase usually occurs in the initial stage of iteration where exploration is more important. Although the velocity ratio is higher than 10, the best strategy for predator in this case is not moving at all. The mathematical model of the high-velocity ratio (v ≥ 10) can be described as:

$$While\;Iter<\frac{1}{3}Max\_Iter$$

$$\vec{{Stepsize}_{i}}=\vec{R_{B}}\times ({Elite}_{i}-\vec{R_{B}}\times \vec{{Prey}_{i}})$$

$$\vec{{Prey}_{i}}=\vec{{Prey}_{i}}+P.\vec{R}\times \vec{{Stepsize}_{i}}$$ (22)

where, $\vec{R_{B}}$ is a vector of random numbers, P = 0.5 is a constant value, and R is a vector of uniform random numbers in the range of [0, 1]. This scenario occurs when either the step size or the velocity of movement is high to achieve high exploration ability in the initial stage of the iterations.

Phase 2: In this case, both predator and prey move at the same velocity in order to search for their own food. This phase is also called the unit velocity ratio. In this phase, the transition from exploration to exploitation occurs which is considered as the intermediate phase of optimization. Thus, both exploration and exploitation happen in this phase, where half of the population is designated for exploration and the rest of the population for exploitation. Notably, the prey is responsible for exploitation while the predator for exploration. In the unit velocity ratio (v ≈ 1), if prey direct in Lévy walk, and Brownian movement will be the best strategy for the predator to attack the prey. This phase can be mathematically expressed as follows:

$$While\;Iter<\frac{1}{3}Max\_Iter<\frac{2}{3}Max\_Iter$$

For the first half of the population

$$\vec{{Stepsize}_{i}}=\vec{R_{L}}\times ({Elite}_{i}-\vec{R_{L}}\times \vec{{Prey}_{i}})$$

$$\vec{{Prey}_{i}}=\vec{{Prey}_{i}}+P.\vec{R}\times \vec{{Stepsize}_{i}}$$ (23)

where, $\vec{R_{L}}$ is a vector of random numbers based on Lévy distribution representing Lévy movement. As exploitation also occurs in the case of the second half of the populations during this phase which can be presented as:

$$\vec{{Stepsize}_{i}}=\vec{R_{B}}\times (R_{B}\times \vec{{Elite}_{i}}-\vec{{Prey}_{i}})$$

$$\vec{{Prey}_{i}}=\vec{{Elite}_{i}}+P.CF\times \vec{{Stepsize}_{i}}$$ (24)

while ${CF=(1-\frac{Iter}{Max\_Iter})}^{\left(2\times \frac{Iter}{Max\_Iter}\right)}$ is considered as an adaptive parameter to control the step size for predator movement. Multiplication of $\vec{R_{B}}$ and Elite simulates the movement of predator in Brownian manner. On the other hand, prey updates its position according to the movement of predators in Brownian motion.

Phase 3: The low-velocity ratio is seen during this phase as the predator is moving faster than prey to attack it which happens in the last phase of the optimization. This low-velocity ratio (v = 0.1) shows the high exploitation ability where the best strategy for the predator is Lévy. This phase can be modeled as:

$$While\;Iter>\frac{2}{3}Max\_Iter$$

$$\vec{{Stepsize}_{i}}=\vec{R_{L}}\times (R_{B}\times \vec{{Elite}_{i}}-\vec{{Prey}_{i}})$$

$$\vec{{Prey}_{i}}=\vec{{Elite}_{i}}+P.CF\times \vec{{Stepsize}_{i}}$$ (25)

As observed from the literature study, the movement of the predator in the Lévy strategy is based on the Multiplication of $\vec{R_{L}}$ and Elite. By adding the step size to the Elite position ensures the movement of the predator to update the prey position. Though, the Lévy and Brownian movement in the whole life span of a predator is the same percentage.

**Fig 1:**
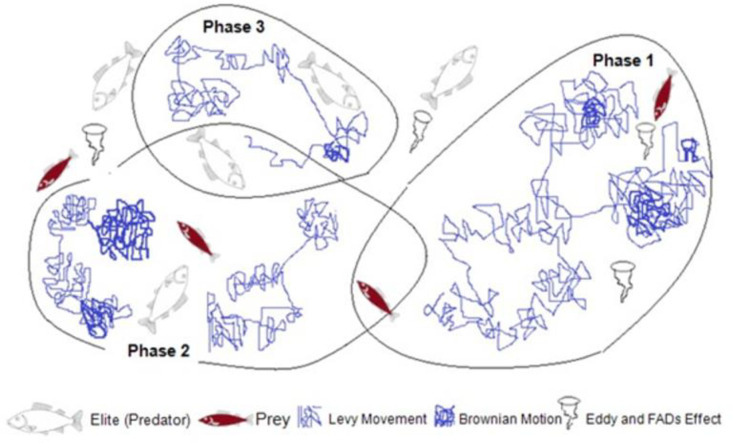
Several optimization phases of the proposed MPA.

In the first stage, the predator is motionless but in the next stage, it moves in Brownian. Besides in the last stage, it shows the Lévy strategy. Since prey is considered as another potential predator for its mimicking behaviour of food. At the first phase of the movement, the prey is moving in Brownian, then in the second phase in the Lévy behavior. Each phase got one-third of the iterations which shows the better-optimized results comparing to the switching or repetition of the strategy. The entire exploration phases of the proposed MPA technique have illustrated in Fig 1.

### 3.3 Eddy formation and FADs’ effect

Environmental factors like eddy formation or Fish Aggregating Devices (FADs) affect the foraging pattern in a marine predator. The FADs are responsible for the local optima and the trapping behaviour in these points in the search region. To avoid such local optima during simulation, this method considers longer jumps. The mathematical representation of FADs is as follows:

$$\vec{{Prey}_{i}}=\left\{ \begin{array}{c} {Prey}_{i}+CF[X_{min}+\vec{R}\times (\vec{X_{max}}-\vec{X_{min}})]\times \vec{U}\;\mathrm{if}\;r\leq {FAD}_{s}\\ \vec{{Prey}_{i}}+[{FAD}_{s}(1-r)+r](\vec{{Prey}_{r1}}-\vec{{Prey}_{r2}})\;\mathrm{if}\;r>{FAD}_{s} \end{array}\right.$$ (26)

where r is the uniform random number in [0, 1], $\vec{X_{min}}$ and $\vec{X_{max}}$ are the vectors containing the lower and upper bounds of the dimensions respectively, r1 and r2 subscripts denote the random indexes of the prey matrix. FADs are the probability of FADs effect and the value is assumed as 0.2 in the optimization which generate a random vector in [0,1]. $\vec{U}$ denotes the binary vector of values zero or one. The array values changes to zero or one if the array value is lesser or greater than 0.2 respectively.

### 3.4 Marine memory

Marine predators have the quality memory that plays an important role in food foraging. Additionally, this memory enhances the capability of the exploration and exploitation in the MPA. The convergence criteria of the Elite are examined after updating the Prey and implementation of the FADs effect. The most fitted potential solution is updated after comparing the immediate solution with respect to the fitness function. Thus, the MPA determines the high-quality solution in the search space.

### 3.5 MPA phases, exploration and exploitation

The exploration and exploitation in the optimization process of MPA can be categorized into three distinct phases. At the first phase of optimization, the prey moves in Brownian motion within the search region. Though the distance between predator and prey is relatively large in Brownian motion but preys can explore their neighbourhood separately in this stage which results in good exploration of the domain. Then, the prey updates the new position after evaluating the fitness function based on the survival theory. Throughout the foraging process, prey can also be replaced as a dominant predator if it shows successful behaviour in food searching.

In the second phase, the algorithm moves from exploration into the exploitation stage. In this case, both prey and predator look for their own food where half of the populations engage for exploration and the other half for exploitation. In this journey, the predator follows the Brownian motion and the prey finds food in the Lévy strategy while in absence of food it takes a long jump in the nearby area. At the end of this phase, predator and prey come closer and the jumping step size decreases, drastically. Additionally, the FADs effect minimizes the possibility of trapping into local optima for better optimization outcome. The foraging behavior switches from Brownian to Lévy strategy for high exploration ability. On the other hand, the search space is restricted by the defined convergence factor (CF) within the search space.

**Fig 2:**
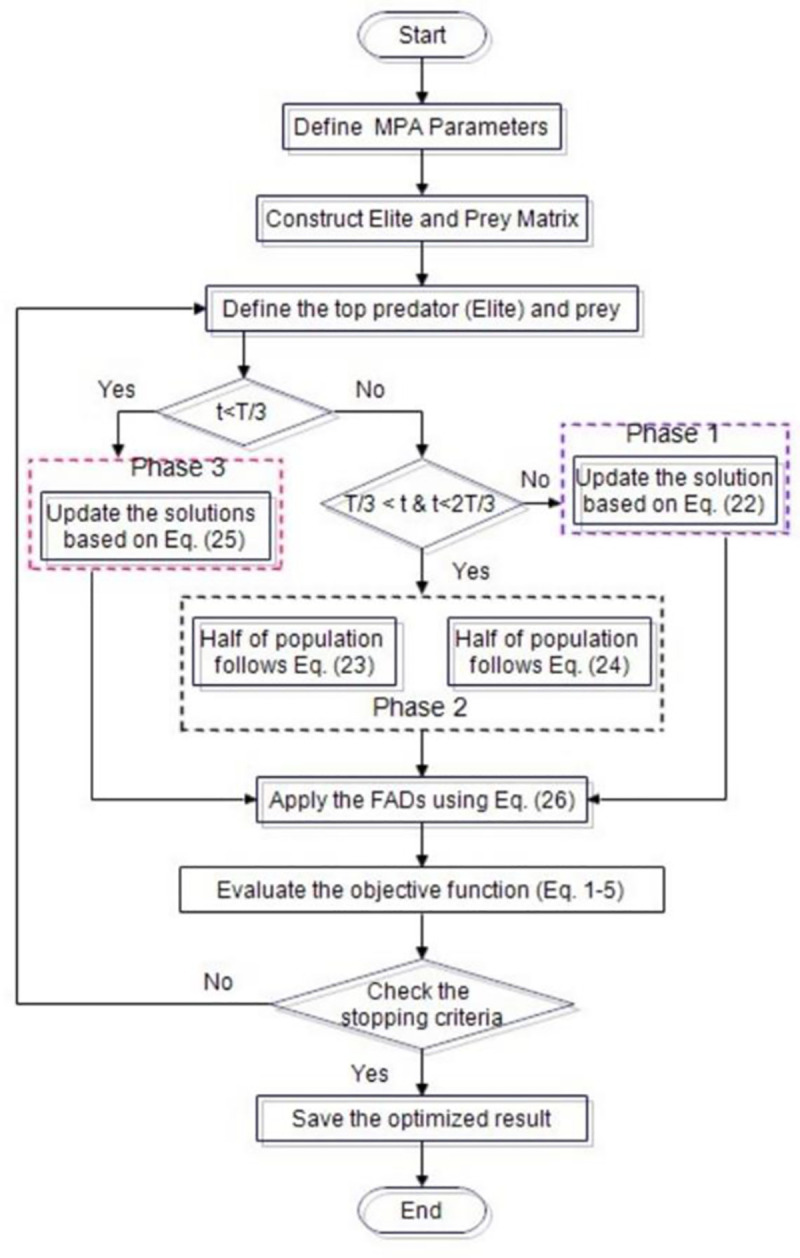
Application of MPA to OPF problem MPA flowchart.

At the last phase, the computational complexity of the proposed method is the minimum and can be depicted as (t (nd + Cof*n)), where t is the number of iteration, n is a number of agents, Cof is the cost of function evaluation, and d is the dimension of the problem to be solved. Fig 2 demonstrates the optimization process of the proposed method in a flowchart.

### 3.6 Application of MPA to the OPF problem

This section presents the step-by-step implementation of MPA in solving OPF problems as described below:

***Step 1***: Input the test system data (e.g., Bus and line data of the system) for the validation purpose.
***Step 2***: Set the MPA parameters such as number of populations, N and total number of iterations, t. The total number of populations will take part to optimize the formulated objective functions in the search space.
***Step 3***: Evaluate the objective functions to be optimized such as fuel cost, active power loss, reactive power loss, voltage deviation and Voltage Stability Enhancement Index considered as single-optimal power flow problems in Eqs 1–5 for each population.
***Step 4***: Now, construct Elite and Prey Matrix in order to get the optimal solution among the populations considered.
***Step 5***: Determine the top predator from elite and prey for updating its position and velocity of the prey for successive iterations.
***Step 6***: Exploration in three phase and update the position using Eqs 22–25
***Step 7***: Apply the FADs effect using Eq 26
***Step 8***: At this step, evaluate the objective function based on Eqs 1–5
***Step 9***: Check the stopping conditions of maximum number of iterations is reached using Eqs 7–18.
***Step 10***: Stop the program if the stopping criteria met, otherwise return to step 2.

## 4. Results and discussion

**Fig 3:**
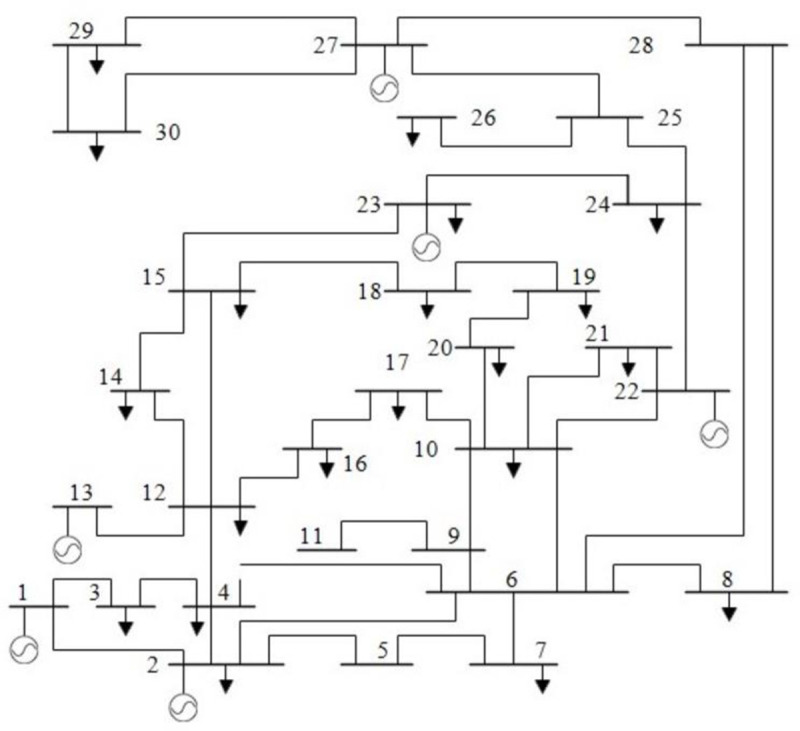
IEEE-30 bus system single line diagram.

The effectiveness and feasibility of the proposed MPA-based optimization method was tested on a standard IEEE 30-bus test system. This test system model consists of six generators bus, four transformers, and nine shunt compensations. The location of the generator at buses 1, 2, 3, 8, 11, and 13. with shunt compensation at buses 10, 12, 15, 17, 20, 21, 23, 24, and 29. Besides, the IEEE 30-bus system has 24 load buses and 41 transmission lines of which 4 branches namely 6–9, 6–10, 4–12, and 28–27 are with the tap setting transformers [38]. It is worth mentioning that this test system has been widely used for OPF study with the maximum load demand of 283.4 MW, in which the total real and reactive power demands are 2.834 pu and 1.262 pu, respectively, with the base MVA of 100. On the other hand, the lower and upper bound limits of transformers tap and load busses were set in the range between [0.9, 1.1] pu and [0.95, 1.05] pu, respectively. Moreover, the minimum and maximum restrictions of the voltage magnitude of the generation units were set as [0.95, 1.1]. The proposed method was coded using MATLAB software in the PC with the subsequent characteristics: Intel core i5, CPU 2.60 GHz, RAM 4GB, and 64-bit operating system. The proposed technique was run with a maximum of 500 iterations and the comparative analysis was carried out for each case of selected objectives as detailed in the forthcoming subsections. The optimal settings of the controlling parameters for the proposed method have also been detailed in Table 1. The IEEE 30-bus system single line diagram has been showed in Fig 3.

**Table 1:** The controlling parameters of IEEE 30-bus system for case 1 to 5 using the MPA based optimization method.

Parameters			Selective Objective		
	FC	Active PL	Reactive PL	VD	VSEI
PG1 (MW)	177.032	51.250	51.309	175.172	171.845
PG2 (MW)	48.688	80	80	48.703	47.874
PG3 (MW)	21.305	50	50	21.515	22.800
PG4 (MW)	21.081	35	35	22.328	23.382
PG5 (MW)	11.912	30	30	12.300	12.808
PG6 (MW)	12.004	40	40	13.184	13.128
V1 (p. u.)	1.1	1.1	1.1	1.035	1.100
V2 (p. u.)	1.088	1.098	1.1	1.019	1.087
V3 (p. u.)	1.062	1.080	1.092	1.010	1.082
V4 (p. u.)	1.069	1.087	1.1	1.001	1.095
V5 (p. u.)	1.1	1.1	1.1	1.062	1.099
V6 (p. u.)	1.1	1.100	1.1	0.997	1.100
T1	1.045	1.057	1.002	1.083	1.021
T2	0.9	0.900	0.966	0.909	0.905
T3	0.987	0.984	0.995	0.956	0.999
T4	0.967	0.973	0.986	0.969	0.981
QC1 (MVAR)	5.000	5.000	5.000	5.000	5
QC2 (MVAR)	5.000	5.000	5.000	0.855	4.998
QC3 (MVAR)	5.000	4.999	4.999	5.000	5.000
QC4 (MVAR)	5.000	5.000	5.000	2.300	5.000
QC5 (MVAR)	5.000	4.999	5.000	5.000	5.000
QC6 (MVAR)	5.000	5.000	5.000	5.000	5.000
QC7 (MVAR)	3.661	3.713	4.999	5.000	5.000
QC8 (MVAR)	5.000	5.000	5.000	5.000	5.000
QC9 (MVAR)	2.995	2.540	3.248	2.722	5.000
Cost ($/h)	**799.0725**	999.8447	967.2060	803.9062	800.3773
Real PL (MW)	8.6223	**2.8513**	2.9102	9.8005	8.4383
Reactive PL (MVAR)	3.0807	24.3630	**-25.2040**	7.2292	5.2224
VD	1.8516	2.0479	2.1310	**0.0992**	2.1264
LK	0.1164	0.1151	0.1142	0.1364	**0.1131**

### 4.1 Case 1 –Fuel cost minimization

**Fig 4:**
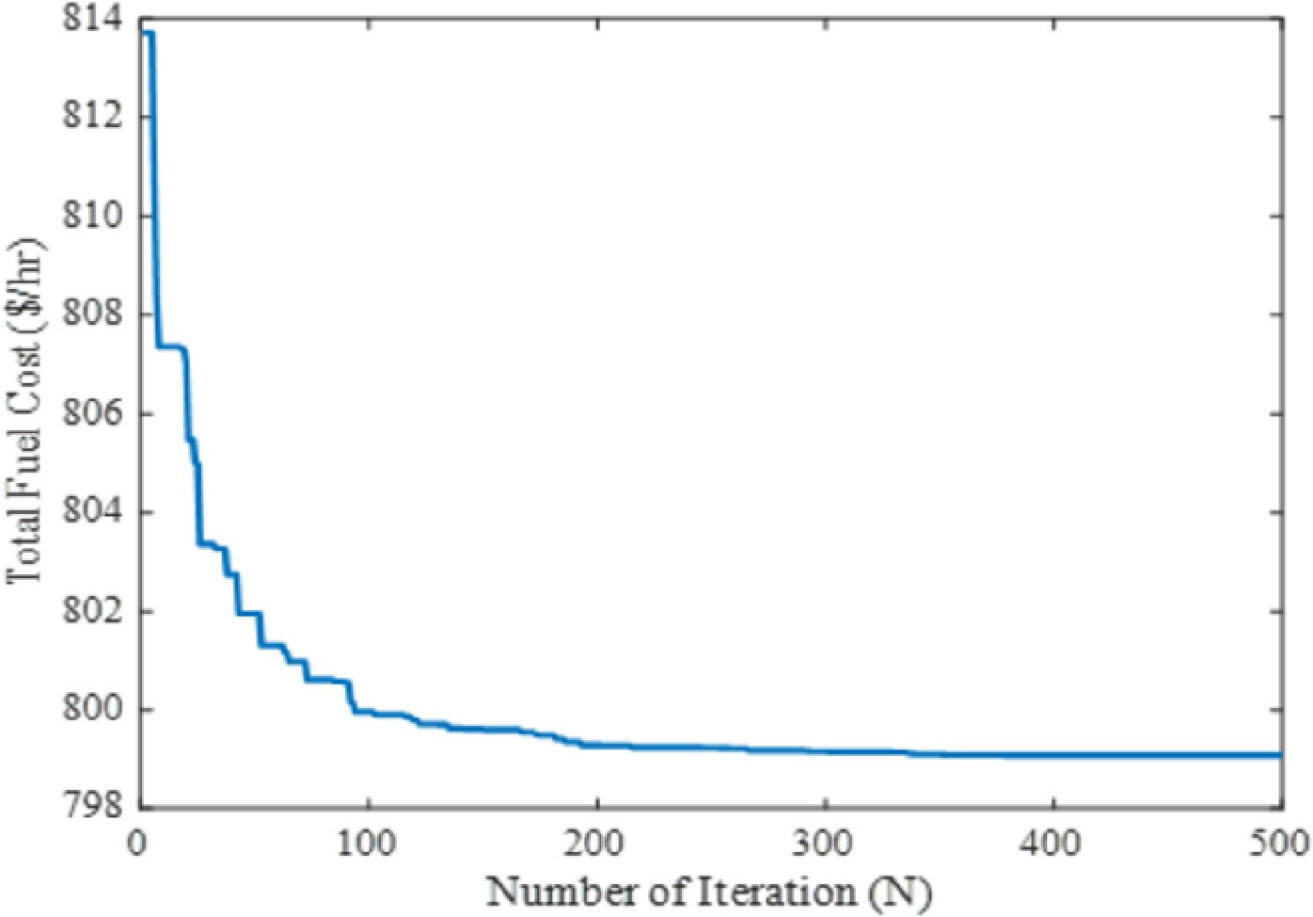
Convergence property of the proposed MPA for case 1.

To verify the effectiveness and performance of the proposed technique in solving the OPF problems, the quadratic fuel cost of each generating unit was considered to optimize as the single-objective function in this case. The mathematical formulation of the objective function is discussed in section 2. The proposed method was employed to analyze all the controlling parameters (i.e., real power generation dispatch) of the IEEE 30-bus test system to meet the required load demand by satisfying the power system constraints. The obtained optimal settings of controlling variables optimize the fuel cost (FC) of the system which is illustrated in Table 2. To generate the least cost power by satisfying all the lower and upper bound restrictions, the generators are initialized randomly in the search region for different iterations. Afterwards, the main optimizer MPA goes through several stages to meet the power demand by enforcing the lower and upper boundaries restriction of each controlling parameter. In the exploration and exploitation stage, the distinctive levy and Brownian movements demonstrated the best global optimum solution in the search space. After the exploitation process, the MPA shows the global optima value at 799.072$/h for fuel cost. The comparison results concerning other metaheuristic-based optimization techniques namely DSA, SCA, MSCA, GWO, DGWO, HAS, FHSA, WEA, EEA, PSO, and DEA reveals that the proposed method showed the global best results among other techniques presented. On the other hand, the DGWO shows the highest value at 801.4333 $/h and is stuck at a certain time. The computational performances in terms of real power generation, real power loss, reactive power loss, voltage deviation, and voltage stability enhancement index for case-1 have been illustrated in Table 2. Thus, from the numerical results, it is seen that the proposed MPA technique provides superior results for the selected single-objective cases among the mentioned literature work. Additionally, the obtained fuel cost using the proposed technique with its convergence characteristics is portrayed in Fig 4.

**Table 2:** Comparison of proposed algorithm with other literature work for case 1.

Parameters	MPA	DSA [39]	SCA [6]	MSCA [6]	GWO [40]	DGWO [40]	HSA [41]	FHSA [41]	WEA [5]	EEA [42]	PSO [43]	DEA [44]
PG1 (MW)	177.032	1.76954	140.21	177.401	171.094	176.949	1.77747	1.76804	1.7706	173.4593	1.7696	176.2592
PG2 (MW)	48.688	0.48713	49.00	48.632	48.615	48.519	0.48584	0.49229	0.48698	47.7363	0.4898	48.5602
PG3 (MW)	21.305	0.21383	20.26	21.2376	21.123	21.326	0.21539	0.21147	0.21302	23.7692	0.2130	21.3402
PG4 (MW)	21.081	0.21285	22.00	20.8615	22.068	21.571	0.21278	0.21043	0.21065	23.2234	0.2119	22.0553
PG5 (MW)	11.912	0.12044	11.00	11.9385	15.479	12.026	0.11014	0.11977	0.11879	11.3724	0.1197	11.7785
PG6 (MW)	12.004	0.12000	11.00	12	13.665	12.001	0.12266	0.12062	0.12	12.2530	0.1200	12.0217
V1 (p.u.)	1.100	1.08442	1.10	1.1	1.080	1.083	1.0951	1.100	1.1	1.0994	1.0855	1.0999
V2 (p.u.)	1.088	1.06454	1.10	1.0867	1.062	1.063	1.0747	1.085	1.0878	1.0853	1.0653	1.0890
V3 (p.u.)	1.062	1.03347	1.08	1.0604	1.030	1.031	1.0410	1.054	1.0618	1.0506	1.0333	1.0659
V4 (p.u.)	1.069	1.03880	1.10	1.0923	1.036	1.035	1.0531	1.062	1.0692	1.0700	1.0386	1.0697
V5 (p.u.)	1.100	1.09793	1.10	1.1	1.080	1.060	1.0976	1.098	1.0909	1.0735	1.0848	1.0965
V6 (p.u.)	1.100	1.04266	1.10	1.1	1.054	1.050	1.0892	1.095	1.1	1.0976	1.0512	1.0996
T1	1.045	1.05000	0.97	1.0439	0.982	0.977	0.9789	1.011	2.69E-0.8	0.9875	1.0233	1.0429
T2	0.900	0.96536	0.95	0.9144	1.026	1.013	0.9395	0.934	0.05	0.9250	0.9557	0.9179
T3	0.987	0.97918	0.96	1.03	0.989	0.934	1.0125	1.008	0.05	1.0375	0.9724	1.0190
T4	0.967	0.97772	0.97	0.9913	0.981	0.975	0.9452	0.976	0.05	1.0250	0.9728	0.9896
QC1 (MVAR)	5.000	0.05000	5.00	0.0246	2.144	1.695	0.0138	0.031	0.043831	0.04	0.0335	4.5453
QC2 (MVAR)	5.000	0.05000	4.80	2.56	2.929	3.394	0.0060	0.045	0.05	0.01	0.0220	4.4158
QC3 (MVAR)	5.000	0.05000	4.99	4.586	1.400	4.777	0.0398	0.041	0.019843	0.05	0.0198	4.1734
QC4 (MVAR)	5.000	0.05000	5.00	2.4098	3.526	4.153	0.0430	0.011	0.039657	0.03	0.0315	2.5171
QC5 (MVAR)	5.000	0.04991	4.60	4.6378	2.954	3.738	0.0346	0.038	0.024189	0.04	0.0454	2.0916
QC6 (MVAR)	5.000	0.05000	4.40	0.3635	3.588	4.941	0.0352	0.013	1.033	0.01	0.0381	4.1990
QC7 (MVAR)	3.661	0.04435	5.00	3.1475	2.974	3.567	0.0002	0.045	0.94417	0.05	0.0398	2.5527
QC8 (MVAR)	5.000	0.05000	5.00	4.8426	3.688	4.996	0.0221	0.023	0.96969	0.03	0.0500	4.3812
QC9 (MVAR)	2.995	0.02992	2.50	3.9411	3.259	2.200	0.0482	0.034	0.95964	0.05	0.0251	2.7503
Cost ($/h)	799.072	800.3887	800.102	799.31	801.259	800.433	800.397	799.914	798.996	800.0831	800.41	799.2891
Real PL (MW)	**8.622**	8.9819	9.0633	8.7327	8.6428	8.9921			8.7613	8.4137	-	8.6150
Reactive PL(MVAR)	3.081	-	-	-	-	-	-	-	-	-	-	-
VD	1.852	-	2.0825	1.4246	0.7285	0.8784	0.0152	0.0146	1.5886	-	0.8765	1.5306
LK	0.116	0.12624	-	-	0.1299	0.1279	-	-	0.1193	0.1303	0.1296	0.1226

### 4.2 Case 2 –Active power loss minimization (APL)

**Fig 5:**
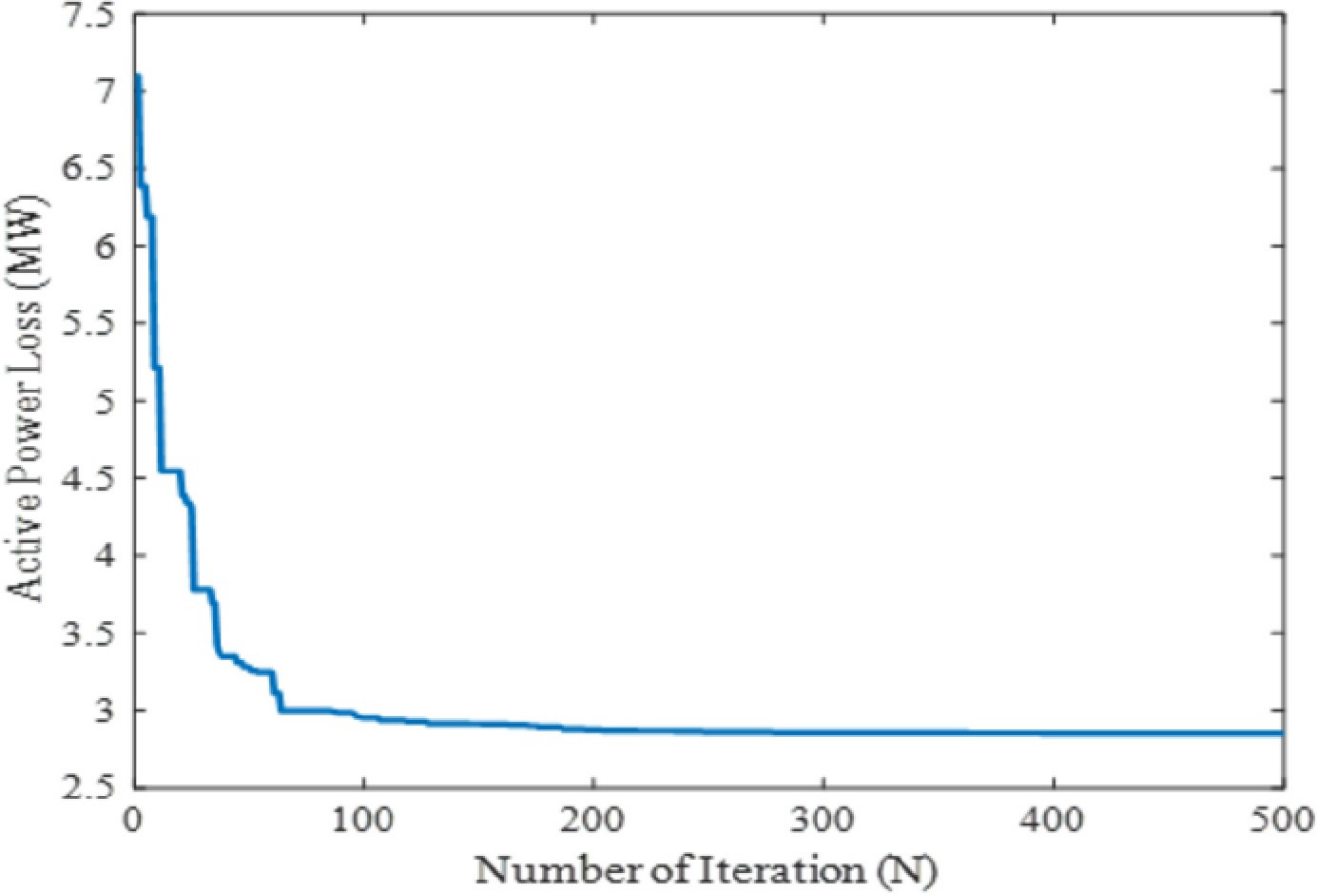
Convergence property of the proposed MPA for case 2.

In this case, to verify the effectiveness and performance of the proposed technique for solving the OPF problems, the active power loss was considered to optimize as the single-objective function. The mathematical formulation of this case is presented in section 2. For this case, the parameter setting for simulation is given in Table 1 and the result was obtained after several phases of exploration and exploitation by the presented approach. After the exploitation process, the MPA shows the global optimal value of 2.8513MW for active power loss. The comparison results with respect to other metaheuristic-based optimization techniques namely DSA, SCA, MSCA, EEA, PSO, ABC, HS and EGA reveals that the proposed method showed the global best results among others in terms of active power loss minimization. On the other hand, the FEA technique shows the highest value at 3.3541 MW while PSO reveals the second highest at 3.318 MW. Although, the modified SCA give the second-best result of 2.9334 MW compared to the original SCA which performs to give 2.9425 MW active power losses. Further, the computational performances in terms of real power generation, real power loss, reactive power loss, voltage deviation, and voltage stability enhancement index for case-2 have been illustrated in Table 3. Thus, from the numerical results, it is seen that the proposed MPA technique provides superior results for the selected single-objective cases among the literature work. Additionally, the obtained power loss for the proffered technique with its convergence characteristics is portrayed in Fig 5.

**Table 3:** Comparison of proposed algorithm with other literature work for case 2.

Parameters	MPA	SCA [6]	MSCA [6]	DSA [39]	PSO [42]	FEA [42]	ABC [39]	HS [39]	EGA [39]
PG1 (MW)	51.250	51.578	52.08	0.510945	56.6613	59.3216	0.510780	0.525327	NR
PG2 (MW)	80	79.78	79.28	0.800000	78.9597	74.8132	0.800000	0.795432	0.80000
PG3 (MW)	50	50.00	50.00	0.500000	49.1795	49.8547	0.500000	0.498152	0.50000
PG4 (MW)	35	34.99	35.00	0.350000	35	34.9084	0.350000	0.347403	0.35000
PG5 (MW)	30	29.99	30.00	0.300000	29.8242	28.1099	0.300000	0.297884	0.30000
PG6 (MW)	40	40.00	39.97	0.400000	37.094	39.7538	0.400000	0.399480	0.40000
V1 (p. u.)	1.100	1.10	1.10	1.0605	1.0694	1.0547	1.0627	1.0754	1.0435
V2 (p. u.)	1.098	1.10	1.07	1.0566	1.0729	1.0418	1.0575	1.0728	1.0435
V3 (p. u.)	1.080	1.08	1.08	1.0378	1.0500	1.0247	1.0385	1.0540	1.0247
V4 (p. u.)	1.087	1.10	1.10	1.0453	1.0476	1.0335	1.0444	1.0637	1.0347
V5 (p. u.)	1.100	1.10	1.10	1.1000	1.0176	1.0229	1.0739	1.0991	1.0700
V6 (p. u.)	1.100	1.10	1.10	1.0474	1.0576	1.0776	1.0463	1.0967	1.0430
T1	1.057	1.01	1.05	1.0329	0.95	1.0125	1.0500	1.0022	1.0375
T2	0.900	0.93	0.95	0.9993	1.0125	0.9125	0.9375	0.9078	0.925
T3	0.984	1.00	1.01	0.9913	0.9875	1.0125	0.9875	0.9593	0.975
T4	0.973	0.97	0.99	0.9786	1.0375	1.0125	0.9750	0.9533	0.975
QC1 (MVAR)	5.000	2.81	3.15	0.0500	0.05	0.04	0.0500	0.0499	0.0500
QC2 (MVAR)	5.000	2.53	0.81	0.0500	0.05	0.02	0.0500	0.0486	0.0300
QC3 (MVAR)	4.999	3.39	4.49	0.0500	0.05	0.05	0.0500	0.0493	0.0000
QC4 (MVAR)	5.000	1.60	2.40	0.0500	0.03	0.01	0.0500	0.0488	0.0100
QC5 (MVAR)	4.999	2.99	1.48	0.0500	0.04	0.05	0.0400	0.0442	0.0400
QC6 (MVAR)	5.000	4.11	4.64	0.0500	0.05	0.00	0.0500	0.0499	0.0200
QC7 (MVAR)	3.713	1.86	3.17	0.0422	0.02	0.02	0.0300	0.0411	0.0500
QC8 (MVAR)	5.000	3.96	4.69	0.0500	0.00	0.05	0.0500	0.0499	0.0500
QC9 (MVAR)	2.540	3.12	1.80	0.0303	0.01	0.02	0.0200	0.0317	0.0500
Cost ($/h)	999.845	966.788	965.648	967.6493	954.348	952.3785	967.681	964.5121	967.86
Real PL (MW)	**2.851**	2.9425	2.9334	3.09450	3.318	3.3541	3.1078	2.9678	3.2008
Reactive PL (MVAR)	-24.363	-	-	-	-	-	-	-	-
VD	2.048	1.8161	1.5987	-	-	-	-	-	-
LK	0.115	-	-	0.12604	-	-	0.1386	0.1154	0.12178

### 4.3 Case 3—Reactive power loss minimization (RPL)

**Fig 6:**
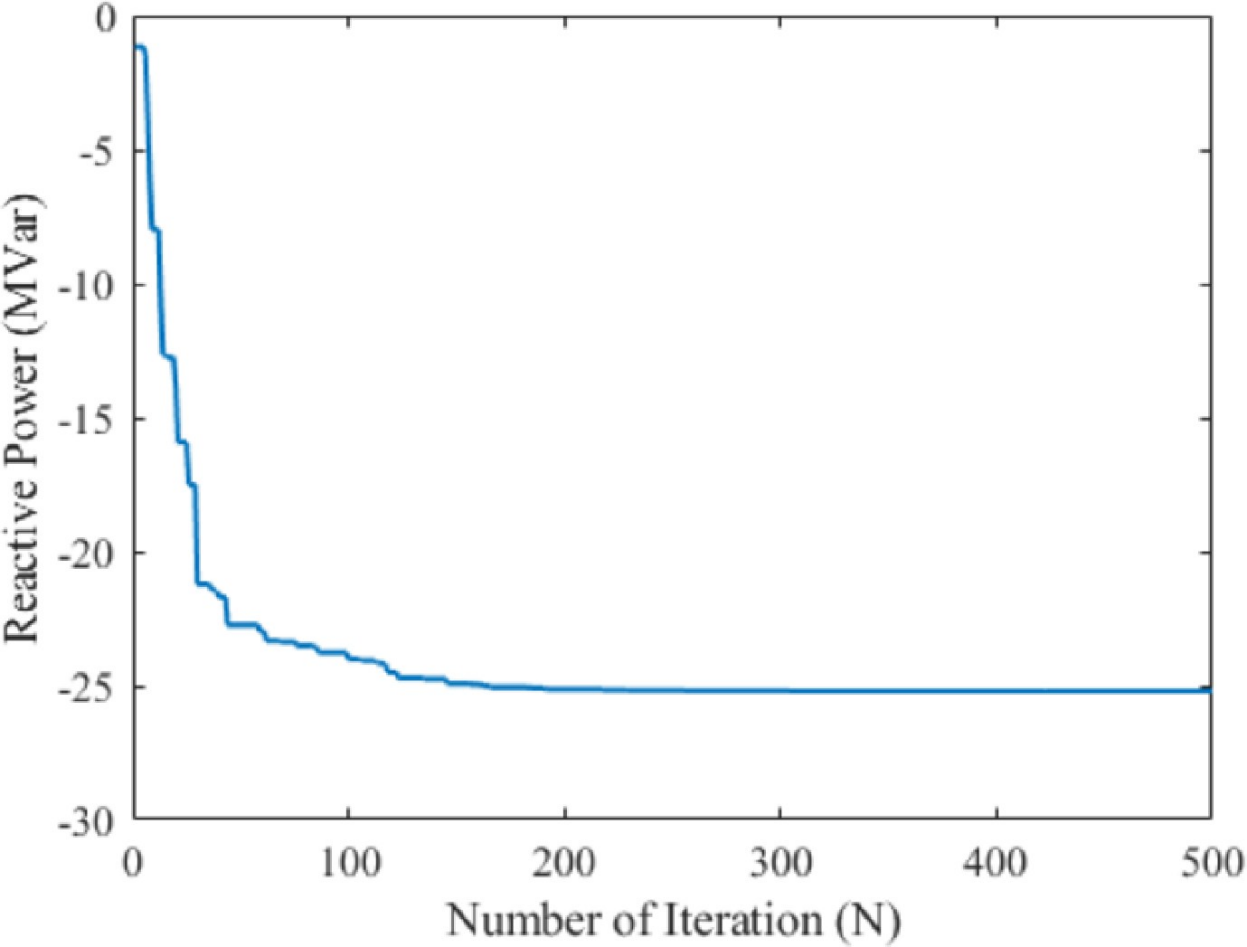
Convergence property of the proposed MPA for case 3.

In this case, further to verify the effectiveness and performance of the propounded technique in solving the OPF problem, the reactive power loss was considered as the single-objective function. The mathematical formulation of this case is also detailed in section 2 with its optimal setting of the control parameter is portrayed in Table 1. The main goal of this objective function is to minimize the reactive power losses by the proposed MPA technique. This objective can be achieved by deducting the reactive power demand from reactive power generation. After the exploitation process, the MPA shows the global optima value of -25.204 MVAR for reactive power loss. The comparison results with respect to other metaheuristic-based optimization techniques reveals that the proposed method showed the global best results among others in the case of reactive power loss minimization. On the other hand, the BHBO technique shows the worst value of -20.1522 MVAR while EM reveals the second- highest value of -22.0196MVAR. Although, the recently used hybrid HFPSO showed almost similar result. The computational performances in terms of real power generation, real power loss, reactive power loss, voltage deviation, and voltage stability enhancement index for case-3 have been illustrated in Table 4. Thus, from the numerical results, it is seen that the proposed MPA technique provides superior results for the selected single-objective cases among all mentioned literature work. Additionally, the obtained reactive power loss with its convergence characteristics is portrayed in Fig 6.

**Table 4:** Comparison of proposed algorithm with other literature work for case 3.

Parameters	MPA	EM [45]	IEM [45]	HFPSO [46]	PSO [46]	BHBO [47]	MVO [48]
PG1 (MW)	51.309	64.0008	51.4349	51.3085	52.0175	73.6130	51.348
PG2 (MW)	80	75.0319	80.0000	80	79.8978	70.9447	80.000
PG3 (MW)	50	48.1465	50.0000	35	49.9998	48.5176	50.000
PG4 (MW)	35	32.7775	35.0000	50	29.8163	31.7662	35.000
PG5 (MW)	30	28.9746	30.0000	35	29.8163	25.5264	29.998
PG6 (MW)	40	37.9527	40.0000	40	40.0000	36.7867	40.000
V1 (p. u.)	1.100	1.0927	1.1000	1.1	1.1000	1.0817	1.100
V2 (p. u.)	1.100	1.0885	1.1000	1.1	1.1000	1.0784	1.100
V3 (p. u.)	1.092	1.0764	1.0939	1.0919	1.0858	1.0651	1.093
V4 (p. u.)	1.100	1.0910	1.1000	1.1	1.1000	1.0703	1.100
V5 (p. u.)	1.100	1.0160	1.1000	1.1	1.0376	1.0088	1.100
V6 (p. u.)	1.100	1.0659	1.1000	1.1	1.0688	1.0398	1.100
T1	1.002	1.0874	1.0121	1.0018	1.0603	1.0504	1.000
T2	0.966	0.9879	0.9000	0.9657	1.0391	0.9973	0.937
T3	0.995	1.0232	0.9870	0.9949	1.0241	1.0104	0.993
T4	0.986	1.0461	0.9816	0.9863	1.0363	1.0284	0.983
QC1 (MVAR)	5.000	3.2124	0.6417	5	0.3746	2.7586	0.775
QC2 (MVAR)	5.000	4.7420	0.0299	5	4.9986	2.5341	3.857
QC3 (MVAR)	4.999	4.1200	4.4270	5	4.9999	2.9776	3.668
QC4 (MVAR)	5.000	1.8437	0.0000	5	1.3503	2.3622	2.923
QC5 (MVAR)	5.000	3.1539	5.0000	5	4.9548	2.9648	4.170
QC6 (MVAR)	5.000	3.2219	4.9813	5	0.6480	2.8198	2.113
QC7 (MVAR)	4.999	4.6849	0.0098	5	2.7229	2.7216	3.390
QC8 (MVAR)	5.000	3.1210	0.0225	5	4.9995	2.7057	5.000
QC9 (MVAR)	3.248	2.8779	4.0354	3.3162	1.4439	2.6123	2.952
Cost ($/h)	967.206	939.4832	967.2229	967.2057	966.95	924.1365	967.250
Real PL (MW)	2.910	3.4851	2.9186	2.9101	2.9101	3.7545	2.948
Reactive PL (MVAR)	**-25.204**	-22.0196	-25.1422	-25.204	-23.756	-20.1522	-25.038
VD	2.1310	0.7773	2.0860	2.1318	0.9126	0.4878	2.041
LK	0.1140	0.1330	0.1162	0.1142	0.1323	0.1371	0.117

### 4.4 Case 4—Voltage deviation (VD)

**Fig 7:**
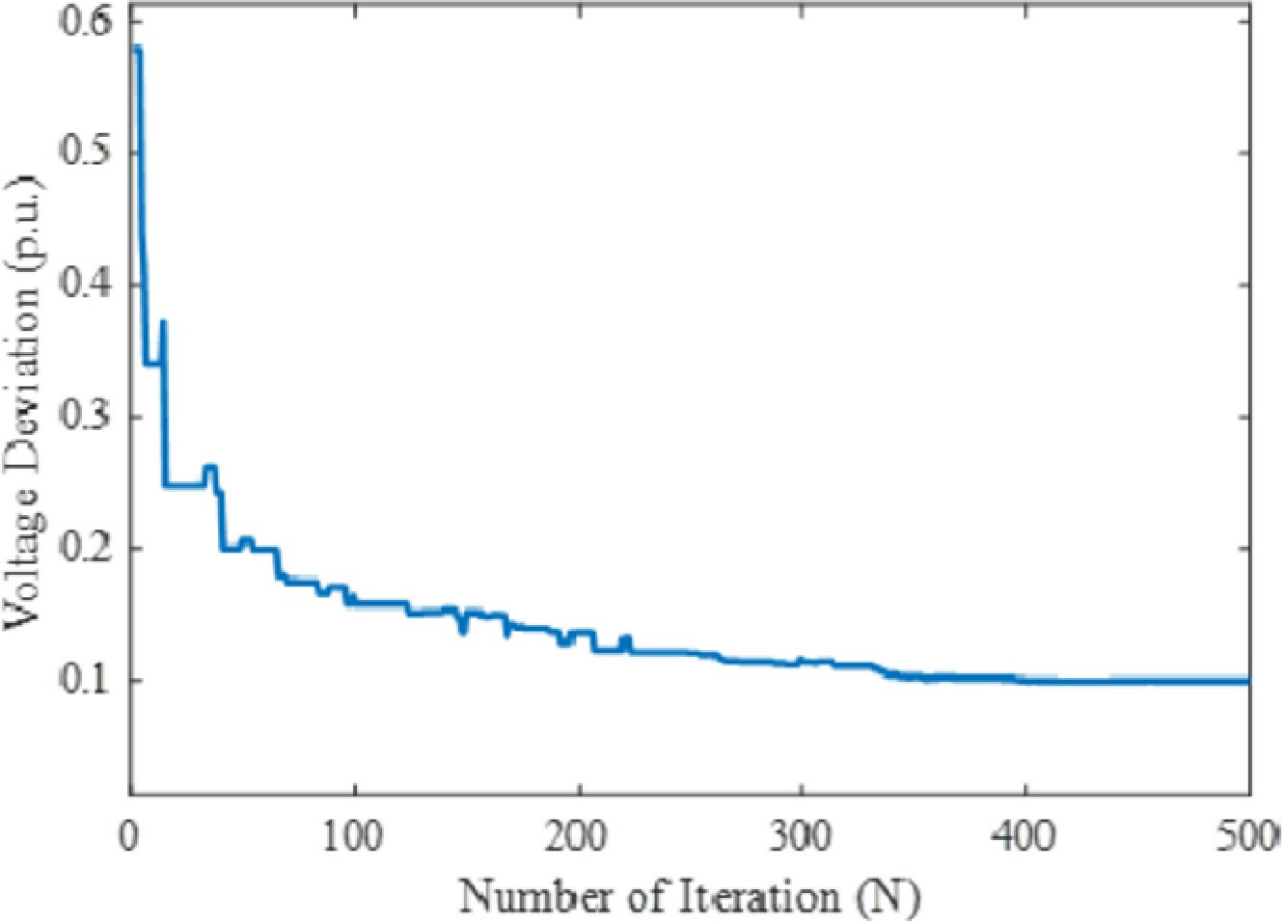
Convergence property of the proposed MPA for case 4.

In this case, the minimization of voltage deviation has been considered to be optimized as the fourth single objective function. The obtained optimal values of all controlling parameters of the power system by the proposed MPA for voltage deviation have been given in Table 4. To verify the effectiveness and performance of the proposed technique in solving the OPF problem comparing with other techniques, the numerical results obtained in the literature from several recently developed meta-heuristic methods have been presented in Table 5. The mathematical formulation of this case is discussed in section 2. At the initial run of the MPA, the algorithm optimizes the parameter by exploring the search space and for the increase in iteration, the exploitation phase increases with a decrease in exploration in order to reach the global optimal solution. After the exploitation process, the MPA shows the global optima value at 0.099 for voltage deviation. The comparison results with respect to other metaheuristic-based optimization techniques namely SCA, MSCA, WEA, PSO, MOJA, and GSA reveal that the proposed method showed the global best results among others. It is observed that all other reported literature work has demonstrated quite similar results in voltage deviation. The computational performances in terms of real power generation, real power loss, reactive power loss, voltage deviation, and voltage stability enhancement index for case-4 have been illustrated in Table 5. Thus, from the numerical results, it is seen that the proposed MPA technique provides superior results for the selected single-objective cases among the presented technique in literature work. Moreover, the optimization of voltage deviation by the proposed technique is given in Fig 7.

**Table 5:** Comparison of proposed algorithm with other literature work for case 4.

Parameters	MPA	SCA [6]	MSCA [6]	WEA [5]	PSO [24]	MOJA [49]	GSA [50]
PG1 (MW)	175.172	122.82	112.585	0.82313	1.7368	89.0808	1.73320940
PG2 (MW)	48.703	74.98	79.76	0.67094	0.4910	78.6206	0.49263900
PG3 (MW)	21.515	15.50	22.25	0.48874	0.2181	49.8306	0.21567799
PG4 (MW)	22.328	31.40	25.09	0.31858	0.2330	34.6289	0.23274500
PG5 (MW)	12.300	29.16	29.95	0.28385	0.1388	23.9941	0.13774500
PG6 (MW)	13.184	18.04	20.85	0.29467	0.1200	12.0077	0.11964300
V1 (p. u.)	1.035	1.00	1.01	1.0055	1.0142	1.0248	1.026900
V2 (p. u.)	1.019	1.04	0.99	1.0028	1.0022	1.0143	1.009980
V3 (p. u.)	1.010	1.02	1.02	1.0191	1.0170	1.0127	1.014280
V4 (p. u.)	1.001	1.04	1.05	1.0037	1.0100	1.0071	1.008680
V5 (p. u.)	1.062	1.00	1.05	0.99844	1.0506	1.0441	1.050289
V6 (p. u.)	0.997	1.03	0.99	1.0047	1.0175	1.0004	1.016340
T1	1.083	0.98	1.04	0.04149	1.0702	1.0646	1.071330
T2	0.909	0.95	0.95	0.03150	0.9000	0.9010	0.900000
T3	0.956	1.00	0.96	0.04979	0.9954	0.9574	0.996500
T4	0.969	0.95	0.95		0.9703	0.9699	0.973200
QC1 (MVAR)	5.000	3.40	4.75	0.04999	0.0403	4.4080	0.04143700
QC2 (MVAR)	0.855	0.09	4.13	0.03514	0.0369	0.0000	0.03562000
QC3 (MVAR)	5.000	2.77	4.87	0.04081	0.0500	4.8290	0.05000000
QC4 (MVAR)	2.300	0.63	3.16	0.05	0.0000	0.0773	0.00000000
QC5 (MVAR)	5.000	4.50	4.93	0.00573	0.0500	4.9988	0.05000000
QC6 (MVAR)	5.000	4.19	4.91	1.0115	0.0500	4.8611	0.05000000
QC7 (MVAR)	5.000	4.79	5.00	0.99173	0.0500	4.9784	0.05000000
QC8 (MVAR)	5.000	4.95	4.93	0.98067	0.0500	4.9206	0.04983700
QC9 (MVAR)	2.722	1.06	0.39	0.95189	0.0259	2.5858	0.02588000
Cost ($/h)	803.906	843.604	8.49.281	911.801	806.38	907.2475	804.314844
Real PL (MW)	9.801	8.5031	7.0828	4.5989	-	4.7626	0.09765939
Reactive PL (MVAR)	7.229	-	-	-	-	-	-
VD	**0.099**	0.1082	0.1030	0.0875	0.0891	0.0935	0.093269
LK	0.136	-	-	0.1262	0.1392	0.1488	0.135776

### 4.5 Case 5—Voltage stability enhancement index (VSEI)

**Fig 8:**
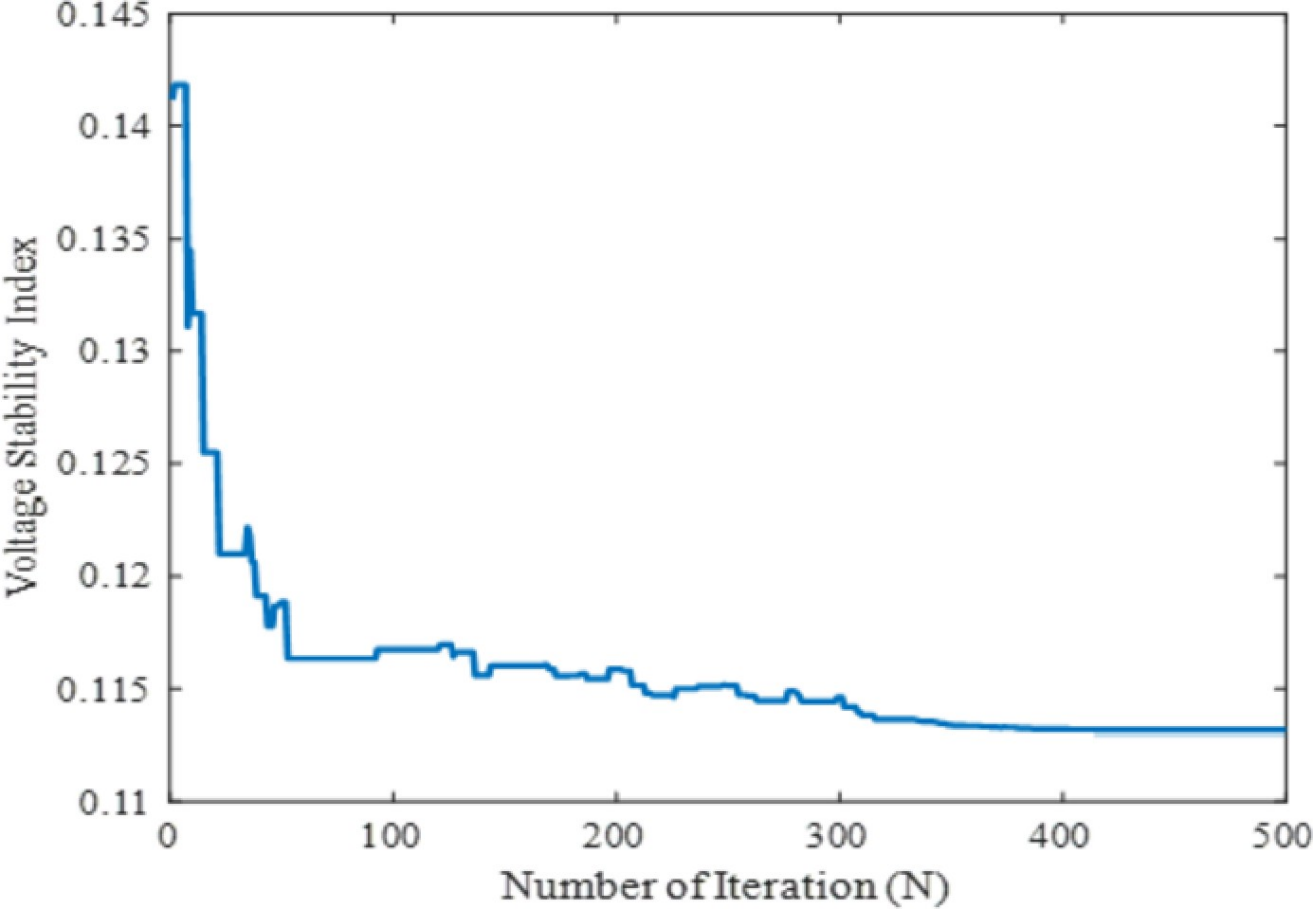
Convergence property of the proposed MPA for case 5.

In this case, to verify the effectiveness and performance of the proposed technique in solving the OPF problem, the voltage stability enhancement index was considered to optimize as the fifth single-objective function. Generally, the voltage stability index should be in the range of zero (no-load case) to one (voltage collapse). This voltage stability index is used to find out the accurate voltage instability of the system in order to avoid the voltage collapse of the power network. Therefore, it is necessary to consider the VSEI in OPF problem- solving. The mathematical formulation of this case is mentioned in section 2. The proposed method was employed to analyze all the controlling parameters of the IEEE 30-bus test system to meet the required demand by satisfying all the power system constraints. The obtained optimal settings of controlling variables to optimize the VSEI of the system which is illustrated in Table 6. In order to ensure the optimized outcomes, the proposed method of MPA undergoes through several stages to meet the power demand by enforcing the lower and upper boundaries restriction of each controlling parameter. In the exploration and exploitation stage, the distinctive levy and Brownian movements demonstrated the best global optimum solution in the search space. After the exploitation process, the MPA shows the global optima value at 0.113 for VSEI. The comparison results with respect to other metaheuristic-based optimization techniques such as WEA, DSA, BBO, MODE, PSO, and GA reveals that the proposed method showed the global best results among others in terms of solution quality and convergence property. On the other hand, the WEA and BBO showed the global minima in case 5 at 0.0927 and 0.09803 respectively, although the other parameters like fuel cost showed the worst value which is are the major concern. The computational performances in terms of real power generation, real power loss, reactive power loss, voltage deviation, and voltage stability enhancement index for case-5 have been illustrated in Table 6. Thus, from the numerical results, it is seen that the proposed MPA technique provides superior results for the selected single-objective cases among all mentioned literature work. Additionally, the convergence characteristic for this case is portrayed in Fig 8.

**Table 6:** Comparison of the proposed algorithm with other literature work for case 5.

Parameters	MPA	WEA [5]	DSA [39]	BBO [39]	MODE [39]	PSO [24]	GA [42]
PG1 (MW)	171.845	1.7874	0.52190	0.99415	0.12712	1.7553	117.971
PG2 (MW)	47.874	0.20106	0.80000	0.34794	0.3885	0.4798	76.13
PG3 (MW)	22.800	0.15001	0.50000	0.49901	0.4476	0.2092	30.99
PG4 (MW)	23.382	0.10003	0.35000	0.34831	0.3400	0.2450	33.43
PG5 (MW)	12.808	0.29992	0.30000	0.29569	0.2669	0.1151	19.099
PG6 (MW)	13.128	0.4	0.39631	0.39995	0.1738	0.1200	13.832
V1 (p. u.)	1.100	1.0998	1.06780	1.0995	1.0700	1.0891	1.04
V2 (p. u.)	1.087	1.0649	1.07250	1.0822	1.0520	1.0693	1.0570
V3 (p. u.)	1.082	1.0017	1.06000	1.0738	1.0610	1.0464	1.0718
V4 (p. u.)	1.095	1.0632	1.05000	1.0499	1.0400	1.0465	1.0223
V5 (p. u.)	1.099	1.1	1.05787	1.0837	1.0980	1.0277	1.0248
V6 (p. u.)	1.100	0.95623	1.01076	0.96403	1.0520	1.0294	1.0450
T1	1.021	0.05	1.0500	1.0999	1.0390	0.9694	0.9250
T2	0.905	0.05	0.9000	1.0999	0.9590	0.9238	0.9125
T3	0.999	0.05	0.9356	1.1000	0.9960	0.9467	0.9000
T4	0.981	0.5	0.9846	0.90246	0.9820	0.9820	1.0750
QC1 (MVAR)	5.000	0.04999	0.0500	0.047741	0.0405	0.0162	0.00
QC2 (MVAR)	4.998	0.5	0.0500	0.049482	0.0442	0.0424	0.05
QC3 (MVAR)	5.000	0.04999	0.0500	0.047491	0.0419	0.0256	0.05
QC4 (MVAR)	5.000	0.5	0.0500	0.047138	0.0498	0.0465	0.03
QC5 (MVAR)	5.000	0.0169	0.0500	0.049353	0.0486	0.0348	0.02
QC6 (MVAR)	5.000	1.1	0.0500	0.049498	0.0490	0.0500	0.03
QC7 (MVAR)	5.000	1.0993	0.0500	0.049404	0.0496	0.0488	0.04
QC8 (MVAR)	5.000	1.1	0.0500	0.048298	0.0490	0.0500	0.05
QC9 (MVAR)	5.000	0.90648	0.0500	0.048054	0.0490	0.0500	0.05
Cost ($/h)	800.377	854.418	967.4718	917.3597	856.90	801.16	844.473
Real PL (MW)	8.438	10.4372	3.4217	4.95	5.40	-	8.052
Reactive PL (MVAR)	5.222	-	-	-	-	-	-
VD	2.126	0.8479	-	-	-	0.9607	-
LK	**0.113**	0.0927	0.1244	0.09803	0.1246	0.1246	0.1133

### 4.6 Case 6—Analysis of large-case test system

**Fig 9:**
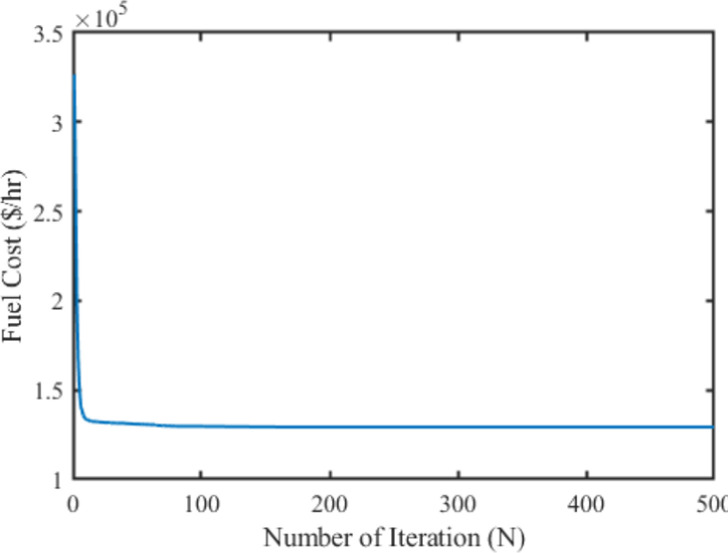
IEEE 118-bus convergence curve.

In this case, an IEEE 118-bus system data has been considered to verify the effectiveness of the proposed technique for solving the large-scale power system. The active and reactive power demand of this system are 4242 MW and 1439 MVAR, respectively. The quadratic fuel cost of each generating unit was considered to be optimize as the single-objective function to demonstrate the effectiveness of proposed method. in this case. The mathematical formulation of this e objective function is discussed in section 2. The proposed method was employed to analyze all the controlling parameters (i.e., real power generation dispatch) of the IEEE 118-bus test system to meet the required load demand by satisfying the power system constraints of equality and inequality. The obtained optimal settings of controlling variables that optimize the fuel cost (FC) of the system which is illustrated in Table 7. To generate the least cost power by satisfying all the lower and upper bound restrictions, the generators are initialized randomly in the search region for different iterations. Afterwards, the main optimizer MPA goes through several stages to meet the power demand by enforcing the lower and upper boundaries restriction of each controlling parameter. In the exploration and exploitation stage, the distinctive levy and Brownian movements demonstrated the best global optimum solution in the search space. After the exploitation process, the MPA shows the global optima value of at 129422.56$/h for fuel cost and active power loss 74.64 MW, respectively. Additionally, the obtained fuel cost using the proposed technique with its convergence characteristics is portrayed in Fig 9.

**Table 7:** Controlling variables of IEEE 118-bus system.

Parameters	Value	Parameters	Value	Parameters	Value	Parameters	Value	Parameters	Value
PG1	25.82641	PG65	4.68592	VG1	1.03644	VG65	1.05635	T8	0.98167
PG4	0.82954	PG66	0	VG4	1.05897	VG66	1.07416	T32	1.0042
PG6	0	PG69	0	VG6	1.05142	VG69	1.08738	T36	0.98786
PG8	0.82235	PG70	16.74587	VG8	1.04061	VG70	1.06097	T51	0.97243
PG10	396.37794	PG72	21.29686	VG10	1.05135	VG72	1.05453	T93	0.99825
PG12	85.63114	PG73	0.38982	VG12	1.04938	VG73	1.06814	T95	0.98374
PG15	18.0197	PG74	425.347	VG15	1.04513	VG74	1.05721	T102	1.03342
PG18	12.1527	PG76	0.2663	VG18	1.04572	VG76	1.04863	T107	0.9885
PG19	20.64721	PG77	4.1874	VG19	1.04579	VG77	1.06263	T127	0.98748
PG24	0	PG80	498.3164	VG24	1.05831	VG80	1.07537	QC5	22.1875
PG25	192.3364	PG85	0	VG25	1.07192	VG85	1.05852	QC34	15.642
PG26	274.8895	PG87	0	VG26	1.08589	VG87	1.08615	QC37	4.2751
PG27	18.35485	PG89	0.4872	VG27	1.05236	VG89	1.07826	QC44	22.3526
PG31	7.17113	PG90	0.3367	VG31	1.04138	VG90	1.06861	QC45	15.2674
PG32	26.24774	PG91	225.6524	VG32	1.045313	VG91	1.07467	QC46	2.3982
PG34	12.85337	PG92	38.4756	VG34	1.06075	VG92	1.07541	QC48	7.826
PG36	7.1522	PG99	0	VG36	1.06205	VG99	1.0673	QC74	11.6733
PG40	33.82485	PG100	7.4375	VG40	1.07354	VG100	1.07719	QC79	24.1432
PG42	31.39974	PG103	34.8527	VG42	1.05268	VG103	1.06346	QC82	5.3876
PG46	18.0887	PG104	6.2875	VG46	1.05152	VG104	1.05618	QC83	7.9287
PG49	193.61117	PG105	147.94211	VG49	1.06358	VG105	1.06313	QC105	14.6295
PG54	49.00784	PG107	0	VG54	1.05604	VG107	1.6288	QC107	6.2836
PG55	31.22416	PG110	347.8255	VG55	1.05829	VG110	1.06429	QC110	22.5176
PG56	55.53385	PG111	35.3524	VG56	1.04872	VG111	1.05344	Fuel Cost ($/h)	129422.56
PG59	149.28334	PG112	30.6253	VG59	1.06105	VG112	1.0611	Active Power Loss (MW)	77.64119
PG61	349.00741	PG113	0	VG61	1.07251	VG113	1.0521		
PG62	462.83747	PG116	0	VG62	1.06084	VG116	1.07613		

## 5. Conclusion

In this work, article, a nature-inspired metaheuristic Marine predator-based optimization technique has been employed to solve several types of single objective OPF problems of fuel cost, real and reactive power loss, voltage deviation and voltage stability enhancement index by satisfying both the equality and inequality constraints of power system network. The effectiveness of the methods is tested on a standard IEEE 30-bus benchmark system and the convergence characteristic exhibits the proposed optimization techniques outperforms to optimal solution. The results obtained for various cases of single-objective function is compared with GA, PSO, BBO, WEA, DSA, and MODE. It is seen that the proposed MPA of fuel cost, active power loss, reactive power loss, voltage deviation, and voltage stability enhancement index was obtained to achieve the global optima of for each individual objective. This is attained through the unique foraging strategy of marine predators with Levy and Brownian movements attributed to getting the competitive optimized results for the formulated OPF problems. The results obtained demonstrated that the proposed method recorded the global minima comparing with other recently developed methods reported in the literature. In particular, the proposed MPA technique showed promising results of 799.0725 $/hr (case-1), 2.851 MW (case-2), -25.204 MVar (case-3), 0.099 (case-4) and 0.113 (case-5) in terms of solution quality for several objectives considered and this claim is also exhibited in convergence characteristics of for different OPF problems studied. Further, to demonstrate the robustness of the proffered technique, an IEEE 118-bus system is tested for the case of fuel cost minimization and the results obtained depicts the optimal fuel cost of 129422.56 $/hr. However, the study of placement of distributed generations and contingency ranking is the future scope of the proposed research work.

## Nomenclature / Abbreviations

ABC: Artificial Bee Colony Algorithm
AI: Artificial Intelligence
APL: Active Power Loss Minimization
BBBC: Big-Bang Big Crunch Algorithm
BBO: Biogeography-based Optimization
BHBO: Black-hole-based Optimization
CF: Convergence Factor
DEA: Differential Evolution Algorithm
DGWO: Developed Grey Wolf Optimizer
DSA: Differential Search Algorithm
EEA: Efficient Evolutionary Algorithm
EGA: Enhanced Genetic Algorithm
EM: Electromagnetism-like Mechanism Algorithm
FADs: Fish Aggregating Devices
FC: Fuel Cost
FHSA: Fuzzy Harmony Search Algorithm
GA: Genetic Algorithm
GSA: Gravitational Search Algorithm
GSO: Glow worm Swarm Optimization Algorithm
GWO: Grey Wolf optimizer
HFPSO: Hybrid Firefly and Particle Swarm Optimization Algorithm
HSA: Harmony Search Algorithm
HSHA: Hybrid Self-adaptive Heuristic Algorithm
IEEE: Institute of Electrical and Electronics Engineers
IEM: Improved Electromagnetism-like Mechanism Algorithm
JA: Jaya Algorithm
MPA: Marine Predator Algorithm
MFO: Moth-Flame Optimization Algorithm
MODE: Multi-objective Differential Evolution Algorithm
MOJA: Multi-objective Jaya Algorithm
MSCA: Modified Sine-Cosine Algorithm
MVO: Multi-verse Optimizer
MVPA: Most Valuable Player Algorithm
OPF: Optimal Power Flow
PL: Power Loss
PSO: Particle Swarm Optimization
RPL: Reactive Power Loss Minimization
SCA: Sine-Cosine Algorithm
TLBO: Teaching-Learning-Based Optimization
TS: Tabu Search Algorithm
VD: Voltage Deviation
VSEI: Voltage Stability Enhancement Index
WEA: Water Evaporation Algorithm
WOA: Whale Optimization Algorithm
**Symbols** u: Control variable
x: Vector of dependent variable
g_j: Equality constraints respectively
h_k: Inequality constraints at kth limit
j, k: j^th and k^th limits.
P_Gslack: Real power generation of slack bus
P_G: Real power generation
Q_G: Reactive power generation
S_L: Transmission line capacity
V_G: Generator voltage bus magnitude
Q_cap: Output of shunt VAR compensator
T: Tap settings of transformers.
a_i, b_i, c_i: Generator cost co-efficient
N_G: Total number of generators
V_k: Voltage amplitude of from bus
V_m: Voltage amplitude of to bus
δ_k: Voltage angle of from bus
δ_m: Voltage angle of to bus
NL: Total number of transmission line
**Algorithm** Xmin: Lower limit of dimensions
Xmax: Upper limit of dimensions
X0: Initial position
X1: Top predator vector
d: Number of dimensions
Xi,j: jth dimension of ith prey
RB: Vector of random numbers
P = 0.5: Constant value
R: Vector of uniform random numbers in the range of [0, 1]
RL: Vector of random numbers based on Lévy movement
U: Binary vector of values 0 or 1
Cof: Cost of function evaluation
